# Supplementary material for: Factors Associated with Low Vitamin D Status among Older Adults in Kuwait
Source: Nutrients. 2022 Aug 15;14(16):3342. doi: 10.3390/nu14163342 (PMC9414672; doi:10.3390/nu14163342)
Supplement: Supplementary file 1 [file nutrients-14-03342-s001.zip › nutrients-1841422-supplementary.pdf]

Supplementary file

Table S1. Socio-demographic characteristics, clinical characteristics and lad data of participants who received/did not receive vitamin D supplementation.

	[ALL]	No supplement	Supplement	OR	P
	N=237	*N=137*	*N=100*		
**Gender:**					
Female	128 (54.0%)	63 (46.0%)	65 (65.0%)	Ref.	Ref.
Male	109 (46.0%)	74 (54.0%)	35 (35.0%)	0.46 [0.27;0.78]	0.004
**Age**	70.0 [67.6;74.3]	70.0 [67.4;74.3]	70.2 [67.9;74.3]	0.99 [0.94;1.05]	0.835
**Dietary intake of vitamin D (IU)**	189 [113;274]	177 [93.7;245]	210 [146;301]	1.00 [1.00;1.00]	0.228
**Dietary intake of Calcium(mg)**	718 [519;1047]	639 [466;950]	805 [610;1117]	1.00 [1.00;1.00]	**0.044**
**Calcium supplement (yes/no)**					
No	230 (97.0%)	135 (98.5%)	95 (95.0%)	Ref.	Ref.
Yes	7 (2.95%)	2 (1.46%)	5 (5.00%)	3.39 [0.68;26.8]	0.139
**Daily calorie intake (Kcal)**	1357 [1040;1727]	1264 [982;1614]	1514 [1097;1880]	1.00 [1.00;1.00]	**0.004**
**Pigmentary Phototype:**					
II/III	154 (65.0%)	92 (67.2%)	62 (62.0%)	1.86 [1.02;3.42]	**0.042**
IV	61 (25.7%)	27 (19.7%)	34 (34.0%)	Ref.	Ref
V	22 (9.28%)	18 (13.1%)	4 (4.00%)	0.34 [0.09;0.98]	**0.045**
**Seasonality**					
Summer/Fall	82 (34.6%)	42 (30.7%)	40 (40.0%)	Ref.	Ref.
Winter/Spring	155 (65.4%)	95 (69.3%)	60 (60.0%)	0.66 [0.39;1.14]	0.140
**Smoke cigarettes**					
No	220 (92.8%)	128 (93.4%)	92 (92.0%)	Ref.	Ref.
Yes	17 (7.17%)	9 (6.57%)	8 (8.00%)	1.24 [0.44;3.41]	0.678
**Comorbidity indicators**					
**Dyslipidemia**					
No	67 (28.3%)	38 (27.7%)	29 (29.0%)	Ref.	Ref.
Yes	170 (71.7%)	99 (72.3%)	71 (71.0%)	0.94 [0.53;1.68]	0.831
**Hypertension**					
No	72 (30.4%)	42 (30.7%)	30 (30.0%)	Ref.	Ref.
Yes	165 (69.6%)	95 (69.3%)	70 (70.0%)	1.03 [0.59;1.82]	0.917
**Cardiovascular disease**					
No	182 (77.1%)	104 (76.5%)	78 (78.0%)	Ref.	Ref.
Yes	54 (22.9%)	32 (23.5%)	22 (22.0%)	0.92 [0.49;1.70]	0.788
**Type 2 Diabetes**					
No	85 (35.9%)	55 (40.1%)	30 (30.0%)	Ref.	Ref.
Yes	152 (64.1%)	82 (59.9%)	70 (70.0%)	1.56 [0.90;2.72]	0.110
**Osteoporosis:**					
No	178 (75.1%)	107 (78.1%)	71 (71.0%)	Ref.	Ref.
Yes	59 (24.9%)	30 (21.9%)	29 (29.0%)	1.45 [0.80;2.64]	0.218
**Weight (Kg)**	77.0 [68.0;86.0]	78.0 [70.0;86.0]	76.0 [66.0;86.0]	0.99 [0.98;1.01]	0.456
**Height (cm)**	161 [155;170]	165 [156;172]	158 [154;166]	0.98 [0.96;1.00]	0.104
**Waist-to-Hip Ratio**	0.94 [0.89;0.98]	0.94 [0.89;0.98]	0.94 [0.88;0.99]	2.23 [0.22;23.1]	0.500
**Waist Hip WHO:**					
Low	67 (28.5%)	46 (34.1%)	21 (21.0%)	Ref.	Ref.
Moderate	40 (17.0%)	20 (14.8%)	20 (20.0%)	2.17 [0.97;4.94]	0.061
High	128 (54.5%)	69 (51.1%)	59 (59.0%)	1.86 [1.01;3.53]	0.048
**BMI catogry**					
Normal weight	30 (12.9%)	15 (11.2%)	15 (15.2%)	Ref.	Ref.
Obese	96 (41.2%)	53 (39.6%)	43 (43.4%)	0.81 [0.35;1.87]	0.624
Overweight	107 (45.9%)	66 (49.3%)	41 (41.4%)	0.62 [0.27;1.42]	0.261
**Sleep Duration_category**					
<6	40 (17.0%)	25 (18.5%)	15 (15.0%)	Ref.	Ref.

6-8	190 (80.9%)	108 (80.0%)	82 (82.0%)	1.26 [0.63;2.60]	0.519
>8	5 (2.13%)	2 (1.48%)	3 (3.00%)	2.40 [0.33;22.6]	0.384
**Sun Exposure**	0.00 [0.00;1.00]	0.00 [0.00;1.00]	0.00 [0.00;3.00]	1.07 [0.98;1.17]	0.127
**Sun Exposure Category:**					
<5 mins	214 (91.1%)	128 (94.8%)	86 (86.0%)	Ref.	Ref.
5-30 mins	21 (8.94%)	7 (5.19%)	14 (14.0%)	2.93 [1.16;8.13]	**0.023**
**Walking per minutes**	4.00 [0.00;7.00]	4.00 [0.00;7.00]	1.00 [0.00;6.00]	0.94 [0.86;1.02]	0.160
**Laboratory test**					
**PTH mmol/L**	5.57 [4.06;7.27]	6.13 [4.64;7.93]	5.02 [3.69;6.51]	0.83 [0.74;0.93]	**0.001**
**PO4 mmol/L**	1.12 [1.02;1.22]	1.09 [1.02;1.19]	1.15 [1.05;1.26]	7.17 [1.41;36.6]	**0.018**
**Ca mmol/L**	2.29 [2.24;2.37]	2.29 [2.22;2.36]	2.32 [2.25;2.38]	5.85 [0.70;48.7]	0.102
**ALP IU/L**	69.0 [57.0;85.0]	68.0 [57.0;80.0]	71.5 [59.8;91.2]	1.01 [1.00;1.02]	**0.027**

Analysis was performed using univariate logistic regression
Continuous normal and non-normal data were summarized using the mean ± SD and median [IQR], respectively
Analysis was performed using Chi-square test of independence for categorical variables
Unpaired t-test and Mann-Whitney test were used to assess the association between vitamin D supplementation and continuous normal and non-normal variables, respectively

Table S2. Results of binomial logistic regression analysis for the factors associated with vitamin D deficiency.

**Predictors**	**Odds Ratios (OR)**	**CI**	**p**
(Intercept)	1.22	0.27 – 5.52	0.796
PTH score	1.16	1.04 – 1.31	**0.016**
**Daily calories (kcal)**	0.9	0.85 – 0.96	**0.001**
**Gender**			
Female	Reference		
Male	2.13	1.16 – 4.02	**0.017**
**Pigmentary phototype**			
IV	Reference		
II/III	0.55	0.28 – 1.07	0.077
V	4.46	1.35 – 20.49	**0.026**
**Seasonality**			
Summer/Fall	Reference		
Winter/Spring	1.67	0.91 – 3.08	0.099
**Sun Exposure**			
<5 mins	Reference		
5-30 mins	0.24	0.08 – 0.70	**0.011**

Table S3. Results of binomial logistic regression analysis for the factors associated with vitamin D deficiency after including vitamin D and calcium intakes.

	vit d def		
Predictors	***Odds Ratios***	*CI*	*p*
(Intercept)	1.93	0.51 – 7.38	0.335
**PTH score**	1.16	1.03 – 1.31	**0.017**
**Daily calories (kcal)**	0.90	0.81 – 0.99	**0.039**
**Gender**			
Female			
Male	2.13	1.15 – 4.05	**0.018**
**Pigmentary phototype**			
IV			
II/III	0.54	0.27 – 1.07	0.077
V	4.49	1.35 – 20.67	**0.026**
**Seasonality**			
Summer/Fall			
Winter/Spring	1.65	0.88 – 3.09	0.116
**Sun exposure category**			
< 5 minutes			
5 – 30 minutes	0.24	0.08 – 0.71	**0.012**
Calcium intake (mg/day)	1.00	1.00 – 1.00	0.942
Vitamin D intake (mg/day)	1.00	1.00 – 1.00	0.864
